# Shared decision making in breast cancer treatment guidelines: Development of a quality assessment tool and a systematic review

**DOI:** 10.1111/hex.13112

**Published:** 2020-08-03

**Authors:** Marta Maes‐Carballo, Isabel Muñoz‐Núñez, Manuel Martín‐Díaz, Luciano Mignini, Aurora Bueno‐Cavanillas, Khalid Saeed Khan

**Affiliations:** ^1^ Department of General Surgery Complexo Hospitalario de Ourense Ourense Spain; ^2^ Department of Preventive Medicine and Public Health University of Granada Granada Spain; ^3^ Department of General Surgery Hospital de Motril Granada Spain; ^4^ Unidad de Mastología de Grupo Oroño Rosario Argentina; ^5^ CIBER of Epidemiology and Public Health (CIBERESP) Madrid Spain; ^6^ Instituto de Investigación Biosanitaria IBS Granada Spain

**Keywords:** breast cancer, breast cancer treatment, clinical practice guidelines, consensus, shared decision making

## Abstract

**Background:**

It is not clear whether clinical practice guidelines (CPGs) and consensus statements (CSs) are adequately promoting shared decision making (SDM).

**Objective:**

To evaluate the recommendations about SDM in CPGs and CSs concerning breast cancer (BC) treatment.

**Search strategy:**

Following protocol registration (Prospero no.: CRD42018106643), CPGs and CSs on BC treatment were identified, without language restrictions, through systematic search of bibliographic databases (MEDLINE, EMBASE, Web of Science, Scopus, CDSR) and online sources (12 guideline databases and 51 professional society websites) from January 2010 to December 2019.

**Inclusion criteria:**

CPGs and CSs on BC treatment were selected whether published in a journal or in an online document.

**Data extraction and synthesis:**

A 31‐item SDM quality assessment tool was developed and used to extract data in duplicate.

**Main results:**

There were 167 relevant CPGs (139) and CSs (28); SDM was reported in only 40% of the studies. SDM was reported more often in recent publications after 2015 (42/101 (41.6 %) vs 46/66 (69.7 %), *P* = .0003) but less often in medical journal publications (44/101 (43.5 %) vs 17/66 (25.7 %), *P* = .009). In CPGs and CSs with SDM, only 8/66 (12%) met one‐fifth (6 of 31) of the quality items; only 14/66 (8%) provided clear and precise SDM recommendations.

**Discussion and conclusions:**

SDM descriptions and recommendations in CPGs and CSs concerning BC treatment need improvement. SDM was more frequently reported in CPGs and CSs in recent years, but surprisingly it was less often covered in medical journals, a feature that needs attention.

## 1 INTRODUCTION

Breast cancer (BC) is the most common cancer in women, with 2.1 million new cases each year (25% of all female cancers), and it also causes the greatest number (about 670000 in 2018, 15%) of cancer‐related deaths among women^1^, ^2^. Mortality and morbidity from BC have decreased in recent years thanks to early diagnosis and the combination of new treatments in a growing array of different strategies^3^, ^4^. The best BC treatment must be personalized^4^, ^5^, and choosing the ideal approach requires a high degree of specialization, scientific‐technical updating, multidisciplinary coordination and patient participation^6^, ^7^, ^8^, ^9^.

This participation in shared decision making (SDM) is considered a keystone in the achievement of sustainable high‐quality cancer care, and it becomes especially important when separate treatment options with overall similar potential can yield very different results depending on patients' preferences^9^, ^10^. In developed countries, SDM is a legal obligation^11^, ^12^, ^13^, and it has been shown to increase the satisfaction of the patient^9^, improve cost‐effectiveness^9^ and reduce malpractice lawsuit^14^. It is claimed to be a keystone to guarantee good quality cancer care^9^, and it is highly recommended by medical associations^15^, ^16^, ^17^.

The implementation of SDM has persistent barriers^18^, ^19^, ^20^, ^21^, ^22^, and it is still poor^23^, ^24^. Many authors have proposed strategies for promotion and practical application of SDM^10^, ^21^, ^25^, ^26^, ^27^, ^28^. A three‐step model introducing choice, describing options and exploring preferences has been suggested^10^. Another proposal involves encouraging patients to make their own care goals that clinicians translate into treatment plans^21^, ^25^. Option Grids and other decision aids are thought to make the SDM process easier^26^, ^27^. Measuring SDM as a quality indicator and reimbursing professionals that actually use SDM have been floated as another idea involving incentivization^28^.

This important subject should be adequately covered in clinical practice guidelines (CPGs) and consensus statements (CSs), especially in those that are published in a medical journal. The aim of this systematic review was to evaluate the characteristics of CPGs and CSs with SDM compared to those without, to develop an SDM quality assessment tool and to collate the specific information and recommendations about SDM concerning BC treatment in women.

## 2 METHODS

This systematic review was carried out following protocol registration (Prospero No: CRD42018106643) and using a prospective protocol developed based on recommended methods for literature searches and assessment of guidelines. During the course of the work, no SDM assessment tool was identified in the literature, so we developed such a tool for data extraction in our work. It was reported according to the preferred reporting items for systematic reviews and meta‐analyses (PRISMA)^29^, ^30^ (see Appendix 1).

### 2.1 Data sources and searches

A systematic search combining MeSH terms "shared decision making", "clinical practice guidelines", "guidelines", "consensus", “breast cancer”, “breast cancer treatment” and including word variants was conducted using MEDLINE covering the period January 2010 to December 2019, without language restrictions. We further searched online databases (EMBASE, Web of Science, Scopus, CDSR, etc.), 12 guideline‐specific databases and 51 websites of relevant professional societies (see Appendix ). For completeness, we searched on the World Wide Web and the bibliographies of known relevant publications to identify additional studies of relevance to the review.

### 2.2 Study selection and data extraction

We included CPGs and CSs about BC management, produced by governmental agencies or national and international professional organizations and societies. We excluded CPGs and CSs about screening and diagnosis, obsolete guidelines replaced by updates from the same organization, and CPG and CSs for education and information purpose only.

Two reviewers (MMC and IMMN) independently considered the potential eligibility of each of the titles and abstracts from the citations and requested full‐text versions. Working independently, reviewers assessed the full text to confirm eligibility. Disagreements were resolved by consensus or arbitration by a third reviewer (MMD). Duplicate articles were identified and removed. Where multiple versions of a CPG or CS were retrieved, the most recent version was reviewed. Data were extracted from selected CPGs and CSs in duplicate, independently. The intraclass correlation coefficient (ICC) was used to assess consistency between reviewers in data extraction, and the reliability level was excellent >0.90^31^. Authoritative guidance^32^ on systematic review methods recommends inter‐reviewer reliability assessment that is designed to compare measurements obtained by two or more reviewers extracting data from the same papers.

### 2.3 Guideline quality assessment and data extraction

We conducted a search to identify a quality assessment tool for SDM. No relevant tools were identified, so we constructed one using consensus to create a checklist from a long list of items identified in the literature searches. The quality of CPGs and CSs for SDM to manage patients with BC was independently evaluated by two different reviewers (MMC and IMMN) using a piloted data extraction form. Disagreements between the two authors (MMC and IMMN) over the risk of bias for particular studies were solved by group discussion involving an arbitrator (MMD) who took the final decision.

### 2.4 Data synthesis

Two authors (MMC and IMMN) synthesized the data extracted to summarize key information within using a piloted data extraction form concerning characteristics of CPGs and CSs with the SDM information and recommendations contained within them. Rate data were compared using chi‐square test to examine whether CPGs and CSs with SDM were different to those without SDM.

## 3 RESULTS

### 3.1 Study selection

**Figure 1:**
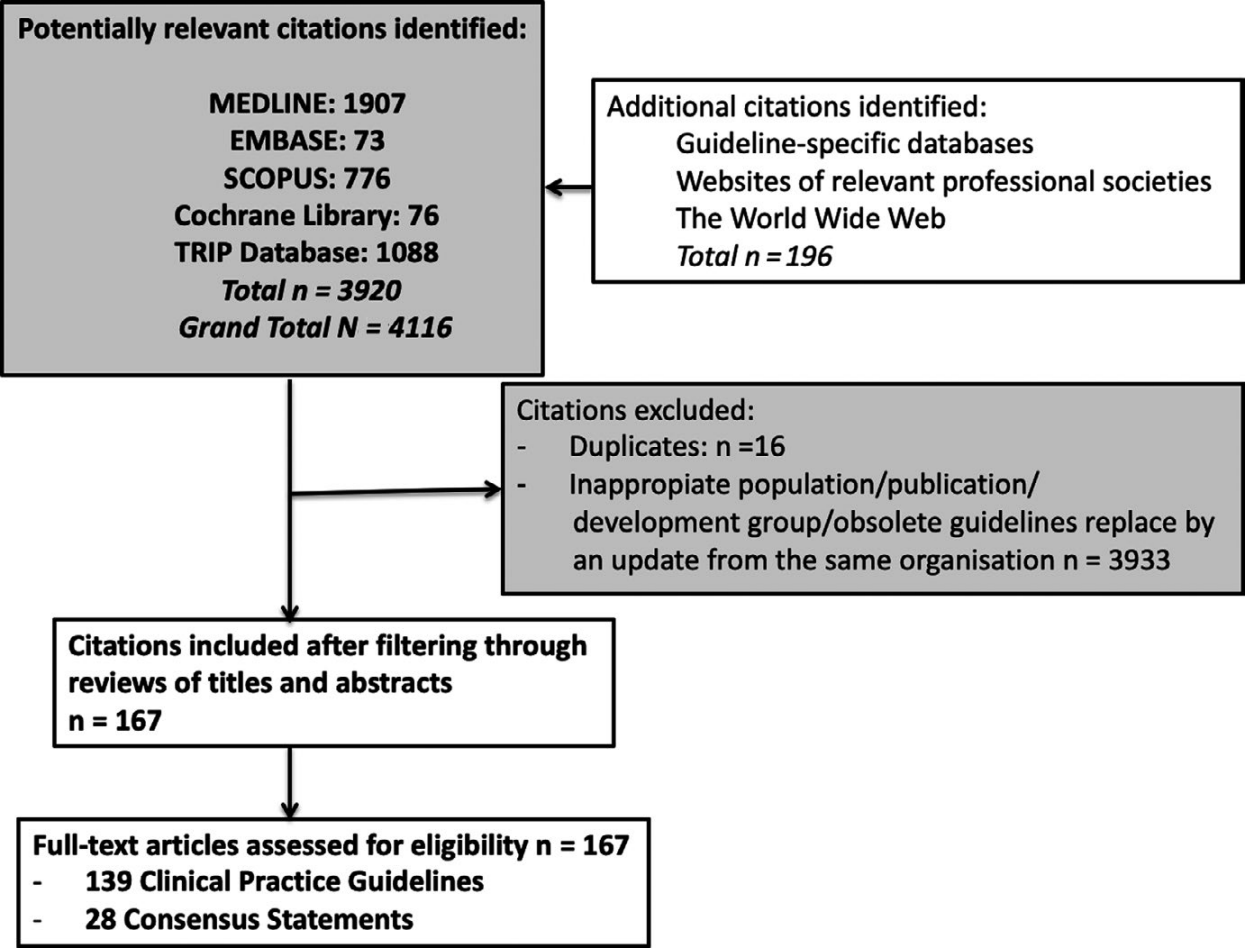
Flow diagram for study selection of CPGs and CSs

Of the 4116 potential citations identified, a total of 167 documents (139 CPGs^33^, ^34^, ^35^, ^36^, ^37^, ^38^, ^39^, ^40^, ^41^, ^42^, ^43^, ^44^, ^45^, ^46^, ^47^, ^48^, ^49^, ^50^, ^51^, ^52^, ^53^, ^54^, ^55^, ^56^, ^57^, ^58^, ^59^, ^60^, ^61^, ^62^, ^63^, ^64^, ^65^, ^66^, ^67^, ^68^, ^69^, ^70^, ^71^, ^72^, ^73^, ^74^, ^75^, ^76^, ^77^, ^78^, ^79^, ^80^, ^81^, ^82^, ^83^, ^84^, ^85^, ^86^, ^87^, ^88^, ^89^, ^90^, ^91^, ^92^, ^93^, ^94^, ^95^, ^96^, ^97^, ^98^, ^99^, ^100^, ^101^, ^102^, ^103^, ^104^, ^105^, ^106^, ^107^, ^108^, ^109^, ^110^, ^111^, ^112^, ^113^, ^114^, ^115^, ^116^, ^117^, ^118^, ^119^, ^120^, ^121^, ^122^, ^123^, ^124^, ^125^, ^126^, ^127^, ^128^, ^129^, ^130^, ^131^, ^132^, ^133^, ^134^, ^135^, ^136^, ^137^, ^138^, ^139^, ^140^, ^141^, ^142^, ^143^, ^144^, ^145^, ^146^, ^147^, ^148^, ^149^, ^150^, ^151^, ^152^, ^153^, ^154^, ^155^, ^156^, ^157^, ^158^, ^159^, ^160^, ^161^, ^162^, ^163^, ^164^, ^165^, ^166^, ^167^, ^168^, ^169^, ^170^, ^171^ and 28 CSs^172^, ^173^, ^174^, ^175^, ^176^, ^177^, ^178^, ^179^, ^180^, ^181^, ^182^, ^183^, ^184^, ^185^, ^186^, ^187^, ^188^, ^189^, ^190^, ^191^, ^192^, ^193^, ^194^, ^195^, ^196^, ^197^, ^198^, ^199^) were identified for final evaluation (Figure 1). ICC for reviewer agreement was 0.97.

### 3.2 Development of a quality assessment tool

Individual quality items were scattered across a number of tools for guidelines assessment ^200^, ^201^. A long list of items was compiled and presented to a group of four BC and SDM specialists in a consensus meeting. This process including several revisions and iterations which led to a 31‐item checklist grouped into thirteen domains (see Appendix ). Of these, 68% (n = 21) were identified from the AGREE^201^ and 48% (n = 15) from the RIGHT^200^ tools. Only 13% (n = 4) of these items did not appear in any of these two tools. However, the expert consensus advised their inclusion after examining other literature in the bibliography of interest about SDM^9^, ^21^, ^24^, ^25^, ^27^. The consensus meeting following approval of the 31‐item checklist recommended that each item be examined for compliance. The greater the percentage of items complied with, the greater the quality for SDM in the CPG or CS assessed. The consensus meeting did not recommend the construction of a formal score or a cut point for defining quality.

### 3.3 Study characteristics

The distribution by countries of CPGs and CSs that speak about SDM was irregular (Figure 1). Europe stood out with a total of 25 CPGs and CSs (38%). North America developed 29 (44%) CPGs and CSs (USA: 19 and Canada: 10). South America released six (9%) CPGs and CSs (Colombia, Venezuela, Mexico, Peru and two from Costa Rica). Asia also carried out three (5%) CPGs and CSs (Japan, India and Malaysia). Oceania has developed also three (5%)CPGs and CSs: two from Australia and one from New Zealand. The basic characteristics of the CPGs and CSs including organization, country and year of release are summarized in Table 1. The duration since last update of each CPGs or CSs varied. Some AGO^46^, ^48^, ^49^, ^59^, all the NCCN^149^, ^150^, ^151^, ^152^, ^153^ and one of the AHS^89^ CPGs, and ESMO^178^ and the Mexican CS^173^ were the most recently updated (highlighted in Table 2). Overall, the last update of the CPGs and CSs with SDM was more recent than that of those without SDM (mean 45 months (range: 3‐115) vs 52 months (range: 3‐116), *P* < .001). In this comparison, 9% (n = 15/167) did not specify the month of updated but only the year. SDM was reported more often in recent CPGs and CSs published after 2015 (42/101 (42.0%) vs 46/66 (69.7%), *P* =.0003) but less often in CPGs and CSs published in medical journal (44/101 (43.5%) vs 17/66 (25.7%), *P* = .009) (Table 3).

**Table 1:** Description of the CPGs and CSs (n = 167) selected for the systematic review on the quality of reporting concerning SDM in BC treatment

		Abbreviated name	Entity	Country	Year
	*Name of the CPG*				
1	Guidelines on the diagnosis and treatment of breast cancer (2011 edition)^32^	Chinese BC CPG^32^	CMH	China	2012
2	Chinese guidelines for diagnosis and treatment of breast cancer 2018^33^	Chinese BC diagnosis treatment^33^	NHCPRC	China	2018
3	The Japanese Breast Cancer Society Clinical Practice Guideline for radiation treatment of breast cancer, 2015 edition^34^	Japanese RT BC CPG^34^	JBCS	Japan	2015
4	The Japanese Breast Cancer Society Clinical Practice Guideline for systemic treatment of breast cancer, 2015 edition^35^	Japanese systemic BC CPG^35^	JBCS	Japan	2015
5	2013 clinical practice guidelines (The Japanese Breast Cancer Society): history, policy and mission^36^	Japanese treatment BC CPG^36^	JBCS	Japan	2014
6	Singapore Cancer Network (SCAN) Guidelines for Adjuvant Trastuzumab Use in Early Stage HER2 Positive Breast Cancer^37^	SCAN early BC^37^	SCAN	Singapore	2015
7	Singapore Cancer Network (SCAN) Guidelines for Bisphosphonate Use in the Adjuvant Breast Cancer Setting^38^	SCAN adjuvant BC treatment^38^	SCAN	Singapore	2015
8	Breast cancer in women: diagnosis, treatment and follow‐up^39^	KCE BC CPG^39^	KCE	Belgium	2015
9	Early breast cancer: ESMO Clinical Practice Guidelines for diagnosis, treatment and follow‐up^40^	ESMO BC 2019^40^	ESMO	Europe	2019
10	International guidelines for management of metastatic breast cancer (MBC) from the European School of Oncology (ESO)^41^	ESO MBC^41^	ESO	Europe	2013
11	The European Society of Breast Cancer Specialists recommendations for the management of young women with breast cancer^42^	EUSOMA 2012^42^	EUSOMA	Europe	2012
12	AGO Recommendations for the Diagnosis and Treatment of Patients with Early Breast Cancer: Update 2019^43^	AGO early BC^43^	AGO	Germany	2019
13	Lesions of Uncertain Malignant Potential (B3) (ADH, LIN, FEA, Papilloma, Radial Scar)^44^	AGO uncertain lesions^44^	AGO	Germany	2019
14	Ductal Carcinoma in Situ (DCIS)^45^	AGO DCIS^45^	AGO	Germany	2019
15	Breast Cancer Surgery Oncological Aspects^46^	AGO oncological^46^	AGO	Germany	2019
16	Oncoplastic and Reconstructive Surgery^47^	AGO oncoplastic^47^	AGO	Germany	2019
17	Adjuvant Endocrine Therapy in Pre‐ and Postmenopausal Patients^48^	AGO adjuvant endocrine^48^	AGO	Germany	2019
18	Adjuvant Cytotoxic and Targeted Therapy^49^	AGO cytotoxic^49^	AGO	Germany	2019
19	Neoadjuvant (Primary) Systemic Therapy^50^	AGO neoadjuvant^50^	AGO	Germany	2019
20	Adjuvant Radiotherapy^51^	AGO RT^51^	AGO	Germany	2019
21	Therapy Side Effects^52^	AGO side effects^52^	AGO	Germany	2019
22	Supportive Care^53^	AGO supportive care^53^	AGO	Germany	2019
23	Breast Cancer: Specific Situations^54^	AGO‐specific situations^54^	AGO	Germany	2019
24	Breast Cancer Follow‐Up^55^	AGO follow‐up^55^	AGO	Germany	2019
25	Loco‐Regional Recurrence^56^	AGO recurrence^56^	AGO	Germany	2019
26	Endocrine and “Targeted” Therapy in Metastatic Breast Cancer^57^	AGO endocrine MBC^57^	AGO	Germany	2019
27	Chemotherapy With or Without Targeted Drugs* in Metastatic Breast Cancer^58^	AGO CT MBC^58^	AGO	Germany	2019
28	Osteooncology and Bone Health^59^	AGO osteooncology^59^	AGO	Germany	2019
29	Specific Sites of Metastases^60^	AGO‐specific MBC^60^	AGO	Germany	2019
30	CNS Metastases in Breast Cancer^61^	AGO CNS MBC^61^	AGO	Germany	2019
31	Complementary Therapy Survivorship^62^	AGO survivorship^62^	AGO	Germany	2019
32	Diagnosis and Treatment of Patients with Primary and Metastatic Breast Cancer^63^	AGO primary MBC^63^	AGO	Germany	2018
33	AGO Recommendations for the Diagnosis and Treatment of Patients with Advanced and Metastatic Breast Cancer: Update 2018^64^	AGO advanced MBC^64^	AGO	Germany	2018
34	DEGRO practical guidelines for radiotherapy of breast cancer VI: therapy of locoregional breast cancer recurrences^65^	DEGRO BC recurrences^65^			2014
35	DEGRO practical guidelines: radiotherapy of breast cancer I. Radiotherapy following breast conserving therapy for invasive breast cancer. ^66^	DEGRO RT conserving BC^66^	DEGRO	Germany	2013
36	DEGRO practical guidelines for radiotherapy of breast cancer IV. Radiotherapy following mastectomy for invasive breast cancer^67^	DEGRO RT mastectomy BC^67^	DEGRO	Germany	2014
37	DEGRO practical guidelines: radiotherapy of breast cancer III—radiotherapy of the lymphatic pathways^68^	DEGRO RT lymphatic^68^	DEGRO	Germany	2014
38	Diagnosis, staging and treatment of patients with breast cancer. National Clinical Guideline No. 7^69^	NCCP^69^	NCCP	Ireland	2015
39	Breast cancer^70^	Richtlijnendatabase BC^70^	Richtlijnen	Netherlands	2018
40	Dutch breast reconstruction guideline^71^	Dutch BCR^71^	DPRS	Netherlands	2017
41	Breast Cancer^72^	IKNL BC^72^	IKNL	Netherlands	2012
42	Cáncer de mama/ Breast Cancer^73^	Fisterra BC^73^	Fisterra	Spain	2017
43	SEOM clinical guidelines in early‐stage breast cancer^74^	SEOM early stage^74^	SEOM	Spain	2018
44	SEOM clinical guidelines in advanced and recurrent breast cancer^75^	SEOM advanced BC^75^	SEOM	Spain	2018
45	SEOM clinical guidelines in metastatic breast cancer^76^	SEOM MBC^76^	SEOM	Spain	2015
46	SEOM clinical guidelines in Hereditary Breast and ovarian cancer^77^	SEOM hereditary BC^77^	SEOM	Spain	2015
47	Abemaciclib with fulvestrant for treating hormone receptor‐positive, HER2‐negative advanced breast cancer after endocrine the therapy^78^	NICE abemaciclib^78^	NICE	UK	2019
48	Ribociclib with fulvestrant for treating hormone receptor‐positive, HER2‐negative advanced breast cancer^79^	NICE ribociclib^79^	NICE	UK	2019
49	Early and locally advanced breast cancer: diagnosis and management^80^	NICE early and advanced BC^80^	NICE	UK	2018
50	Breast cancer^81^	NICE BC^81^	NICE	UK	2011
51	Familial breast cancer: classification, care and managing breast cancer and related risks in people with a family history of breast cancer^82^	NICE familial BC^82^	NICE	UK	2013
52	Breast reconstruction using lipomodelling after breast cancer treatment^83^	NICE lipomodelling^83^	NICE	UK	2012
53	Gene expression profiling and expanded immunohistochemistry tests for guiding adjuvant chemotherapy decisions in early breast cancer management: MammaPrint, Oncotype DDX,X, IHC4 and Mammostrat^84^	NICE gene expression^84^	NICE	UK	2013
54	Pertuzumab for the neoadjuvant treatment of HER2‐positive breast cancer^85^	NICE pertuzumab BC^85^	NICE	UK	2016
55	Intraoperative tests (RD‐100i OSNA system and Metasin test) for detecting sentinel lymph node metastases in breast cancer^86^	NICE sentinel lymph^86^	NICE	UK	2013
56	Breast reconstruction following prophylactic or therapeutic mastectomy for breast cancer^87^	AHS reconstruction BC^87^	AHS	Canada	2017
57	Adjuvant systemic therapy for early stage (lymph node negative and lymph node positive) breast cancer^88^	AHS early BC^88^	AHS	Canada	2018
58	Optimal use of taxanes in metastatic breast cancer (MBC)^89^	AHS MBC^89^	AHS	Canada	2013
59	Adjuvant radiation therapy for invasive breast cancer^90^	AHS RT invasive^90^	AHS	Canada	2015
60	Adjuvant radiation therapy for ductal carcinoma in situ^91^	AHS RT DCI^91^	AHS	Canada	2015
61	Neo‐adjuvant (pre‐operative) therapy for breast cancer ‐ general considerations ^92^	AHS neo‐adjuvant^92^	AHS	Canada	2014
62	The Role of Trastuzumab in Adjuvant and Neoadjuvant Therapy in Women with HER2/neu‐overexpressing Breast Cancer^93^	CCO trastuzumab Her2 + BC^93^	CCO	Canada	2011
63	Surgical management of early‐stage invasive breast cancer^94^	CCO surgical management BC ^94^	CCO	Canada	2015
64	Breast irradiation in women with early stage invasive breast cancer following breast conserving surgery^95^	CCO RT^95^	CCO	Canada	2016
65	The role of the taxanes in the management of metastatic breast cancer^96^	CCO taxane MBC^96^	CCO	Canada	2011
66	Vinorelbine in stage IV breast cancer^97^	CCO vinorelbine^97^	CCO	Canada	2012
67	The role of aromatase inhibitors in the treatment of postmenopausal women with metastatic breast cancer^98^	CCO aromatase inhibitor MBC^98^	CCO	Canada	2012
68	Epirubicin, as a single agent or in combination, for metastatic breast cancer^99^	CCO epirubicin MBC^99^	CCO	Canada	2011
69	Adjuvant taxane therapy for women with early‐stage, invasive breast cancer^100^	CCO taxane adjuvant therapy BC^100^	CCO	Canada	2011
70	Adjuvant systemic therapy for node‐negative breast cancer^101^	CCO sQT for node‐negative BC^101^	CCO	Canada	2011
71	Adjuvant ovarian ablation in the treatment of premenopausal women with early stage invasive breast cancer^102^	CCO ovarian ablation early stage^102^	CCO	Canada	2010
72	The role of gemcitabine in the management of metastatic breast cancer^103^	CCO gemcitabine^103^	CCO	Canada	2011
73	The role of trastuzumab (herceptin) in the treatment of women with Her2/neu‐overexpressing metastatic breast cancer^104^	CCO trastuzumab MBC^104^	CCO	Canada	2010
74	Capecitabine in stage IV breast cancer^105^	CCO capecitabine^105^	CCO	Canada	2011
75	The role of her2/neu in systemic and radiation therapy for women with breast cancer^106^	CCO her2/neu and RT treatment ^106^	CCO	Canada	2012
76	Locoregional therapy of locally advanced breast cancer (LABC)^107^	CCO LABC^107^	CCO	Canada	2014
77	The role of taxanes in neoadjuvant chemotherapy for women with non‐metastatic breast cancer^108^	CCO taxane neoadjuvant therapy^108^	CCO	Canada	2011
78	Optimal systemic therapy for early female breast cancer^109^	CCO early BC^109^	CCO	Canada	2014
79	Use of adjuvant bisphosphonates and other bone‐modifying agents in breast cancer^110^	CCO bone‐modifying agent BC^110^	CCO	Canada	2016
80	The Role of Aromatase Inhibitors in Adjuvant Therapy for Postmenopausal Women with Hormone Receptor‐positive Breast Cancer^111^	CCO aromatase inhibitors HR + ^111^	CCO	Canada	2012
81	Margin width in breast conservation Surgery^112^	ABS margin width BC^112^	ABS	UK	2015
82	Antibiotic prophylaxis in breast surgery^113^	ABS AB prophylaxis^113^	ABS	UK	2015
83	Management of The malignant axilla In early breast cancer^114^	ABS axila BC^114^	ABS	UK	2015
84	Breast operation note Documentation^115^	ABS BC^115^	ABS	UK	2015
85	Update on optimal duration of adjuvant antihormonal therapy^116^	ABS antihormonal therapy^116^	ABS	UK	2015
86	Oncoplastic breast reconstruction^117^	ABS/BAPRAS oncoplastic^117^	ABS, BAPRAS	UK	2012
87	Acellular dermal matrix (ADM) assisted breast reconstruction procedures^118^	ABS/BAPRAS ADM^118^	ABS, BAPRAS	UK	2012
88	Breast Cancer Clinical Quality Performance Indicators^119^	SCT quality indicators^119^	SCT	UK	2016
89	Treatment of primary breast cancer^120^	SIGN^120^	SIGN	UK	2013
90	Lipomodelling Guidelines for Breast Surgery^121^	JGBSA lipomodelling^121^	JGBSA	UK	2012
91	Performance and Practice Guidelines for the Use of Neoadjuvant Systemic Therapy in the Management of Breast Cancer^122^	ASBS NaQT BC^122^	ASBS	USA	2017
92	Performance and Practice Guidelines for Mastectomy^123^	ASBS mastectomy^123^	ASBS	USA	2014
93	Performance and Practice Guidelines for Breast‐Conserving Surgery/Partial Mastectomy^124^	ASBS breast conserving^124^	ASBS	USA	2014
94	Performance and Practice Guidelines for Axillary Lymph Node Dissection in Breast Cancer Patients^125^	ASBS ALD^125^	ASBS	USA	2014
95	Performance and Practice Guidelines for Sentinel Lymph Node Biopsy in Breast Cancer Patients^126^	ASBS SLND^126^	ASBS	USA	2014
96	Evidence‐Based Clinical Practice Guideline: Autologous Breast Reconstruction with DIEP or Pedicled TRAM Abdominal Flaps^127^	ASPS DIEP and TRAM^127^	ASPS	USA	2017
97	Use of Endocrine Therapy for Breast Cancer Risk Reduction: ASCO Clinical Practice Guideline Update^128^	ASCO endocrine therapy risk BC^128^	ASCO	USA	2019
98	Postmastectomy Radiotherapy: An American Society of Clinical Oncology, American Society for Radiation Oncology, and Society of Surgical Oncology Focused Guideline Update^129^	ASCO postmastectomy RT^129^	ASCO	USA	2017
99	Breast Cancer Surveillance Guidelines^130^	ASCO surveillance^130^	ASCO	USA	2013
100	Selection of Optimal Adjuvant Chemotherapy and Targeted Therapy for Early Breast Cancer: ASCO Clinical Practice Guideline Focused Update^131^	ASCO treatment for early BC^131^	ASCO	USA	2018
101	Systemic Therapy for Patients With Advanced Human Epidermal Growth Factor Receptor 2–Positive Breast Cancer: ASCO Clinical Practice Guideline Update^132^	ASCO systemic therapy EGR2 BC^132^	ASCO	USA	2018
102	Recommendations on Disease Management for Patients With Advanced Human Epidermal Growth Factor Receptor 2–Positive Breast Cancer and Brain Metastases: ASCO Clinical Practice Guideline Update^133^	ASCO EGRF2 MBC^133^	ASCO	USA	2018
103	Integrative Therapies During and After Breast Cancer Treatment: ASCO Endorsement of the SIO Clinical Practice Guideline^134^	ASCO BC treatment^134^	ASCO	USA	2018
104	Chemotherapy and Targeted Therapy for Women With Human Epidermal Growth Factor Receptor 2–Negative (or unknown) Advanced Breast Cancer: American Society of Clinical Oncology Clinical Practice Guideline^135^	ASCO EGFR2 advanced BC^135^	ASCO	USA	2014
105	Role of Bone‐Modifying Agents in Metastatic Breast Cancer: An American Society of Clinical Oncology–Cancer Care Ontario Focused Guideline Update^136^	ASCO bone‐modifying agent MBC^136^	ASCO	USA	2017
106	Recommendations for Human Epidermal Growth Factor Receptor 2 Testing in Breast Cancer: American Society of Clinical Oncology/College of American Pathologists Clinical Practice Guideline Update^137^	ASCO EGFR2 recommendations^137^	ASCO	USA	2013
107	Breast Cancer Follow‐Up and Management After Primary Treatment: American Society of Clinical Oncology Clinical Practice Guideline Update^138^	ASCO follow‐up/management BC^138^	ASCO	USA	2013
108	Adjuvant Endocrine Therapy for Women With Hormone Receptor–Positive Breast Cancer: American Society of Clinical Oncology Clinical Practice Guideline Update on Ovarian Suppression^139^	ASCO ovarian suppression BC^139^	ASCO	USA	2016
109	Role of Patient and Disease Factors in Adjuvant Systemic Therapy Decision Making for Early‐Stage, Operable Breast Cancer: American Society of Clinical Oncology Endorsement of Cancer Care Ontario Guideline Recommendations^140^	ASCO factors in early BC^140^	ASCO	USA	2016
110	Use of Adjuvant Bisphosphonates and Other Bone‐Modifying Agents in Breast Cancer: A Cancer Care Ontario and American Society of Clinical Oncology Clinical Practice Guideline^141^	ASCO use bone‐modifying agents BC^141^	ASCO	USA	2017
111	Use of Biomarkers to Guide Decisions on Adjuvant Systemic Therapy for Women With Early‐Stage Invasive Breast Cancer: American Society of Clinical Oncology Clinical Practice Guideline Focused Update^142^	ASCO biomarkers in early BC^142^	ASCO	USA	2017
112	Use of Biomarkers to Guide Decisions on Systemic Therapy for Women With Metastatic Breast Cancer: American Society of Clinical Oncology Clinical Practice Guideline^143^	ASCO biomarkers in MBC^143^	ASCO	USA	2019
113	American Society of Clinical Oncology Endorsement of the Cancer Care Ontario Practice Guideline on Adjuvant Ovarian Ablation in the Treatment of Premenopausal Women With Early‐Stage Invasive Breast Cancer^144^	ASCO ovarian ablation BC^144^	ASCO	USA	2011
114	American Society of Clinical Oncology/College of American Pathologists Guideline Recommendations for Immunohistochemical Testing of Estrogen and Progesterone Receptors in Breast Cancer^145^	ASCO hormonal BC^145^	ASCO	USA	2010
115	Use of Pharmacologic Interventions for Breast Cancer Risk Reduction: American Society of Clinical Oncology Clinical Practice Guideline^146^	ASCO risk reduction BC^146^	ASCO	USA	2013
116	Endocrine Therapy for Hormone Receptor–Positive Metastatic Breast Cancer: American Society of Clinical Oncology Guideline^147^	ASCO endocrine BC^147^	ASCO	USA	2016
117	Invasive Breast Cancer. Basic resources. Version 1.2019^148^	NCCN invasive BC basic^148^	NCCN	USA	2019
118	Invasive Breast Cancer. Core resources. Version 1.2019^149^	NCCN invasive BC core^149^	NCCN	USA	2019
119	Invasive Breast Cancer. Enhanced resources. Version 1.2019^150^	NCCN invasive BC enhanced^150^	NCCN	USA	2019
120	Breast Cancer. NCCN Evidence Blocks. Version 1.2019^151^	NCCN evidence block BC^151^	NCCN	USA	2019
121	Breast Cancer. Version 3.2019^152^	NCCN BC^152^	NCCN	USA	2019
122	Management of Breast Cancer (2nd Edition)^153^	MHM BC^153^	MHM	Malaysia	2010
123	Influencing best practice in breast cancer^154^	Australia BC^154^	AG	Australia	2016
124	Recommendations for staging and managing the axilla^155^	CA axilla^155^	CA	Australia	2011
125	Recommendations for use of hypofractionated radiotherapy for early operable breast cancer^156^	CA RT^156^	CA	Australia	2011
126	Recommendations for use of Bisphosphonates^157^	CA bisphosphonates^157^	CA	Australia	2011
127	Recommendations for the management of early breast cancer in women with an identified BRCA1 or BRCA2 gene mutation or at high risk of a gene mutation^158^	CA management BC^158^	CA	Australia	2014
128	Guía de Práctica Clínica AUGE Cáncer de Mama^159^	GPC Chile^159^	MSC	Chile	2015
129	Guía de práctica clínica (GPC) para la detección temprana, tratamiento integral, seguimiento y rehabilitación del cáncer de mama^160^	GPC Colombia^160^	INC	Colombia	2017
130	Guía de Práctica Clínica del Tratamiento para el Cáncer de Mama^161^	GPC Costa Rica^161^	IHCAI	Costa Rica	2011
131	Guía de Práctica Clínica para el Tratamiento del Cáncer de Mama ^162^	GPC Perú^162^	DDSS	Perú	2017
132	Guía para el Cáncer de Mama en Venezuela^163^	GPC Venezuela^163^	SAV	Venezuela	2015
133	Management of Early Breast Cancer^164^	New Zealand BC^164^	MHNZ	New Zealand	2014
134	The Screening, Diagnosis, Treatment, and Follow‐Up of Breast Cancer^165^	Würzburg BC^165^	UHW	Germany	2018
135	Breast cancer brain metastases: a review of the literature and a current multidisciplinary management guideline^166^	FESEO brain MBC^166^	FESEO	Spain	2013
136	Cirugía de la Mama^167^	AEC BC^167^	AEC	Spain	2017
137	NCA Breast Cancer Clinical Guidelines^168^	NCA BC^168^	NCA	UK	2019
138	Breast Cancer: Management and Follow‐Up^169^	BCMA management and follow‐up^169^	BCMA	Canada	2013
139	Clinical Guidelines for the Management of Breast Cancer^170^	WMCA BC ^170^	WMCA	UK	2016
	*Name of the CS*				
140	Consenso costarricense sobre prevención, diagnóstico y tratamiento del cáncer mamario^171^	CS Costa Rica^171^	CMCCR	Costa Rica	2016
141	Consenso Mexicano sobre diagnóstico y tratamiento del cáncer mamario ^172^	GPC México^172^	SSM	México	2019
142	National consensus in China on diagnosis and treatment of patients with advanced breast cancer^173^	Chinese BC CS^173^	CECM	China	2015
143	Practical consensus recommendations for hormone receptor‐positive Her2‐negative advanced or metastatic breast cancer^174^	Indian ICON CS^174^	ICON	India	2013
144	Indian Solutions for Indian Problems—Association of Breast Surgeons of India (ABSI) Practical Consensus Statement, Recommendations, and Guidelines for the Treatment of Breast Cancer in India^175^	Indian ABSI CS^175^	ABSI	India	2017
145	Consensus document for management of breast cancer^176^	Indian ICMR CS^176^	ICMR	India	2016
146	4th ESO–ESMO International Consensus Guidelines for Advanced Breast Cancer (ABC 4)^177^	ABC4^177^	ESMO	Europe	2018
147	St. Gallen/Vienna 2019: A Brief Summary of the Consensus Discussion about Escalation and De‐Escalation of Primary Breast Cancer Treatment^178^	St. Gallen 2019^178^	St. Gallen	Europe	2019
148	ESTRO consensus guideline on target volume delineation for elective radiation therapy of early stage breast cancer^179^	ESTRO RT BC^179^	ESTRO	Europe	2014
149	Second international consensus guidelines for breast cancer in young women (BCY2)^180^	BCY2^180^	ESO	Europe	2016
150	Guidelines for diagnostics and treatment of aromatase inhibitor‐induced bone loss in women with breast cancer A consensus of Lithuanian medical oncologists, radiation oncologists, endocrinologists, and family medicine physicians^181^	LOEGP^181^	LOEGP	Lithuania	2014
151	Biomarkers in breast cancer: A consensus statement by the Spanish Society of Medical Oncology and the Spanish Society of Pathology^182^	SEOM and SEAP^182^	SEOM	Spain	2017
152	Provincial consensus recommendations for adjuvant systemic therapy for breast cancer^183^	CCM 2017^183^	CCM	Canada	2017
153	Postoperative radiotherapy for breast cancer: UK consensus statements^184^	RCR postoperative RT^184^	RCR	UK	2016
154	Consensus Guideline on Accelerated Partial Breast Irradiation^185^	ASBS RT^185^	ASBS	USA	2018
155	Consensus Guideline on the Use of Transcutaneous and Percutaneous Ablation for the Treatment of Benign and Malignant Tumors of the Breast^186^	ASBS ablation^186^	ASBS	USA	2018
156	Consensus Guideline on the Management of the Axilla in Patients With Invasive/In‐Situ Breast Cancer^187^	ASBS axilla^187^	ASBS	USA	2019
157	Consensus Guideline on Breast Cancer Lumpectomy Margins^188^	ASBS margins^188^	ASBS	USA	2017
158	Consensus Guideline on Concordance Assessment of Image‐Guided Breast Biopsies and Management of Borderline or High‐Risk Lesions^189^	ASBS borderline lesions^188^	ASBS	USA	2016
159	Contralateral Prophylactic Mastectomy (CPM) Consensus Statement from the American Society of Breast Surgeons: Data on CPM Outcomes and Risks^190^	ASBS CPM^190^	ASBS	USA	2016
160	Consensus Guideline on Venous Thromboembolism (VTE) Prophylaxis for Patients Undergoing Breast Operations^191^	ASBS VTE prophylaxis BC^191^	ASBS	USA	2011
161	The American Brachytherapy Society consensus statement on intraoperative radiation therapy^192^	AB intraoperative RT^192^	AB	USA	2017
162	The American Brachytherapy Society consensus report for accelerated partial breast irradiation using interstitial multicatheter brachytherapy ^193^	AB partial RT BC^193^	AB	USA	2017
163	Society of Surgical Oncology Breast Disease Working Group Statement on Prophylactic (Risk‐Reducing) Mastectomy^194^	SSO prophylactic mastectomy^194^	SSO	USA	2016
164	SSO‐ASTRO Consensus Guideline on Margins for Breast‐Conserving Surgery with Whole‐Breast Irradiation in Ductal Carcinoma In Situ^195^	SSO margins ^195^	SSO	USA	2016
165	SSO‐ASTRO Consensus Guideline on Margins for Breast‐Conserving Surgery with Whole Breast Irradiation in Stage I and II Invasive Breast Cancer^196^	SSO–ASTRO invasive BC^196^	SSO ‐ ASTRO	USA	2014
166	Margins for Breast‐Conserving Surgery With Whole‐Breast Irradiation in Stage I and II Invasive Breast Cancer: American Society of Clinical Oncology Endorsement of the Society of Surgical Oncology/American Society for Radiation Oncology Consensus Guideline^197^	ASCO margin BC CSs^197^	ASCO	USA	2014
167	International expert panel on inflammatory breast cancer: consensus statement for standardized diagnosis and treatment^198^	International expert panel BC^198^	IEP	International	2010

**Table 2:** Characteristics of the CPGs and CSs regarding SDM

Characteristics	CPGs or CSs without SDM (n = 101)	CPGs or CSs with SDM (n = 66)	*P* value
Published after 2015	42 (42.0 %)	46 (69.7 %)	.0003
CPG	83 (82.1 %)	54 (81.8 %)	.95
European guidelines	45 (44.5 %)	25 (37.0 %)	.21
North American guidelines	43 (42.5 %)	28 (42.4 %)	.98
South American guidelines	2 (1.9 %)	5 (7.5 %)	.1
Asia guidelines	9 (8.9 %)	3 (4.5 %)	.15
Oceania guidelines	3 (2.9 %)	3 (4.5 %)	.3
Published in a journal	44 (43.5 %)	17 (25.7 %)	.009

**Table 3:** Update frequency of each CPGs/CSs where SDM appears

		Entity	First year of publication	2010	2011	2012	2013	2014	2015	2016	2017	2018	2019
	**CPGs**												
3	Japanese RT BC CPG^34^	JBCS	2015						*				
9	ESMO BC 2019^40^	ESMO	2010	*					*				*
11	EUSOMA 2012^42^	EUSOMA	2012			*							
12	**AGO early BC** ^43^	**AGO**	**2012**			*	*	*	*				*
14	**AGO DCIS** ^45^	**AGO**	**2002**	*		*	*	*	*	*	*	*	*
16	**AGO oncoplastic** ^47^	**AGO**	**2012**				*	*	*	*	*	*	*
17	**AGO adjuvant endocrine** ^48^	**AGO**	**2012**			*	*	*	*	*	*	*	*
27	**AGO CT MBC** ^58^	**AGO**	**2012**			*	*	*	*	*	*	*	*
41	IKNL BC^72^	IKNL	2008			*							
42	Fisterra BC^73^	Fisterra	2011		*						*		*
47	NICE abemaciclib^78^	NICE	2019										*
48	NICE ribociclib^79^	NICE	2019										*
49	NICE early and advanced BC^80^	NICE	2018									*	
50	NICE BC^81^	NICE	2011		*								
51	NICE familial BC^82^	NICE	2013				*						
52	NICE lipomodelling^83^	NICE	2012			*							
53	NICE gene expression^84^	NICE	2013				*						
54	NICE pertuzumab BC^85^	NICE	2016							*			
56	AHS reconstruction BC^87^	AHS	2013				*				*		
57	**AHS early BC** ^88^	**AHS**	**2014**					*	*	*		*	
63	CCO surgical management BC ^94^	CCO	1996		*				*				
70	CCO sQT for node‐negative BC^101^	CCO	1998	*									
71	CCO ovarian ablation early stage^102^	CCO	2010	*									
73	CCO trastuzumab MBC^104^	CCO	1999		*								
76	CCO LABC^107^	CCO	2014					*					
79	CCO bone‐modifying agents BC^110^	CCO	2016							*			
86	ABS/BAPRAS oncoplastic^117^	ABS, BAPRAS	2012			*							
88	SCT quality indicators^119^	SCT	2016							*			
98	ASCO postmastectomy RT^129^	ASCO	2001					*			*		
100	ASCO treatment for early BC^131^	ASCO	2016							*		*	
104	ASCO EGFR2 advanced BC^135^	ASCO	2014					*					
105	ASCO bone‐modifying agent MBC^136^	ASCO	2000		*						*		
108	ASCO ovarian suppression BC^139^	ASCO	2016							*			
109	ASCO factors in early BC^140^	ASCO	2019										
110	ASCO use bone‐modifying agent BC^141^	ASCO	2017								*		
116	ASCO endocrine BC^147^	ASCO	2016							*			
117	**NCCN invasive BC basic** ^148^	**NCCN**	**2015**							*		*	*
118	**NCCN invasive BC core** ^149^	**NCCN**	**2015**							*		*	*
119	**NCCN invasive BC enhanced** ^150^	**NCCN**	**2015**							*		*	*
120	**NCCN evidence block BC** ^151^	**NCCN**	**2015**						*	*		*	*
121	**NCCN BC** ^152^	**NCCN**	**2015**							*		*	*
122	MHM BC^153^	MHM	2002	*									
123	Australia BC^154^	AG	2016							*			
124	CA axilla^155^	CA	2011		*								
129	GPC Colombia^160^	INC	2013				*				*		
130	IHCAI GPC Costa Rica^161^	IHCAI	2011		*								
131	GPC Perú^162^	IETSI	2017								*		
132	GPC Venezuela^163^	SAV	2015						*				
133	New Zealand BC^164^	MHNZ	2009					*					
134	Würzburg BC^165^	UHW	2018									*	
136	AEC BC^167^	AEC	2007								*		
137	NCA BC^168^	NCA	2019										*
138	BCMA management and follow‐up ^169^	BCMA	2013				*						
139	WMCA BC ^170^	WMCA BC	2016										
	**CSs**												
140	CS Costa Rica^171^	CMCCR	2016										
**141**	**GPC México** ^172^	**SSM**	**1994**		*		*		*		*		*
145	Indian ICMR CS^176^	ICMR	2016							*			
**146**	**ABC4** ^177^	**ESMO**	**2012**			*		*		*		*	
147	St. Gallen 2019^178^	St. Gallen	2015						*		*		*
152	CCM 2017^183^	CCM	2017								*		
154	ASBS RT^185^	ASBS	2018									*	
156	ASBS axilla^187^	ASBS	2019										*
158	ASBS borderline lesions^189^	ASBS	2016							*			
159	ASBS CPM^190^	ASBS	2016							*			
163	SSO prophylactic mastectomy^194^	SSO	2007							*			
164	SSO margins^195^	SSO	2014					*					

### 3.4 SDM in CPGs and CSs concerning BC

**Figure 2:**
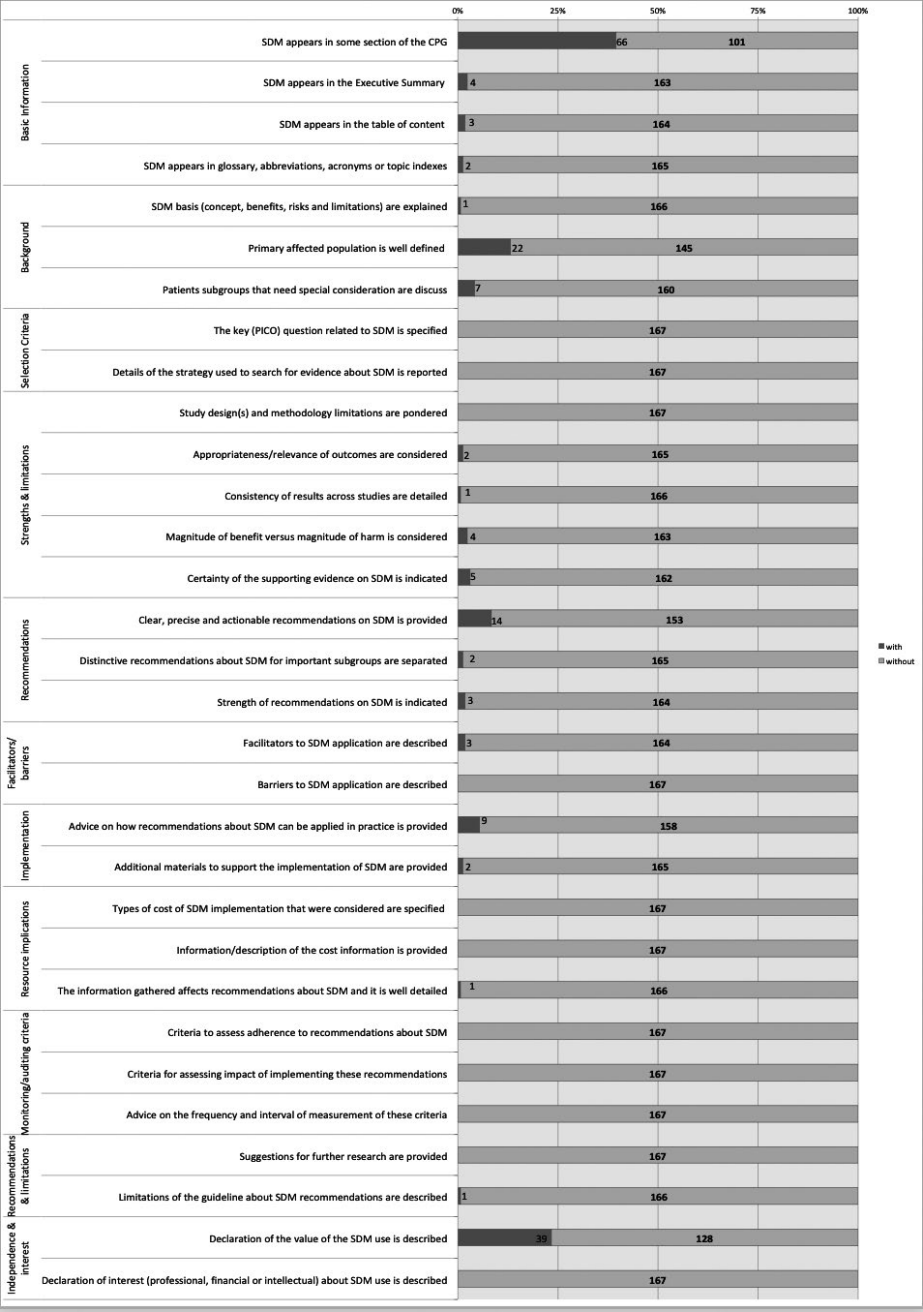
The analysis of the compliance of the data extraction items

The analysis of the compliance of the items valued is presented in Figure 2 and Appendix 4. SDM appeared in any section of 66 CPGs and CSs (12/28 (43%) CSs vs 54/139 (39%) CPGs, *P* = .69). SDM appeared in glossary or indexes in only two documents, and only in one, its basis was explained. In general, CSs had higher overall quality than CPGs (CSs' mean 2.833 vs CPGs' mean 1.12 items, *P* < .001) (Appendix ).

Overall, 39 (23%) stated the value of SDM as an option in the decision‐making process, 14 (8%) provided clear and precise SDM recommendations, 4 (3%) considered benefits versus harms of using SDM, and 4 (2%) identified evidence supporting the use of SDM. Only 9 (5%) of these CPGs and CSs gave advice for the SDM application in practice. The strength of recommendations on SDM was indicated in three (2%). Support for the implementation of SDM was well‐detailed in two documents (1%). The information gathered about SDM affected recommendations and was detailed in one (<1%). Limitations of the CPG or CS about SDM recommendations were described in just one of them (<1%).

Only 4 (2%) of these guides emphasized their interest in SDM appearing in the executive summary. Only in three (2%) of the CPGs and CSs, the table of content talked about SDM. Primary affected population with BC was well‐defined in 22 (13%) articles, and patients’ subgroups with special consideration were discussed in 7 (4%) documents. Appropriateness and relevance of outcomes were considered in only 2 (1%) CPGs. Only one document detailed the consistency of results across studies. Recommendations about SDM for subgroups were separated in only two articles (1%). Facilitators and barriers to SDM application were described in only two articles too (1%).

Ten items (32%) measured in the data extraction instrument were not included in any CPGs and CSs (n = 10/31). The PICO question related to SDM was not specified, search strategy was not reported, the study design and limitations were not pondered, barriers were not described, the cost of SDM implementation was not specified, adherence to recommendations and the impact were not assessed, description of the cost information and suggestions for further research were not provided and finally, professional, financial or intellectual interest about SDM was not described (Figure 2 and Appendix ). Finally, there were 101 (61%) CPGs or CSs did not talk about SDM.

All three reviewers categorized that the 'Alberta Health Services'^88^, 'Australian Government'^155^, 'Ministry of Health from New Zealand'^165^ and Costa Rica 'IHCAI'^162^ CPGs and 'CMCCR'^172^ CS had the highest overall quality in analysing the decision‐making process in BC treatment (Appendix ). In the United States of America, we highlighted two of the 'American Society of Clinical Oncology (ASCO)'^140^, ^141^, ^142^, ^143^, ^144^, ^145^, ^146^, ^147^, ^148^ guidelines and the last version of NCCN^153^, but with a lower mark if you compare with the ones we named before. In Europe, we found the 'European Society for Medical Oncology (ESMO)'^41^, the 'Asociación Española de Cirujanos (AEC)'^80^ and the 'ABS‐BAPRAS'^118^ CPGs with a score of 6 as the best paradigm of a guide that talks about SDM.

## 4 DISCUSSION

### 4.1 Main findings

**Figure 3:**
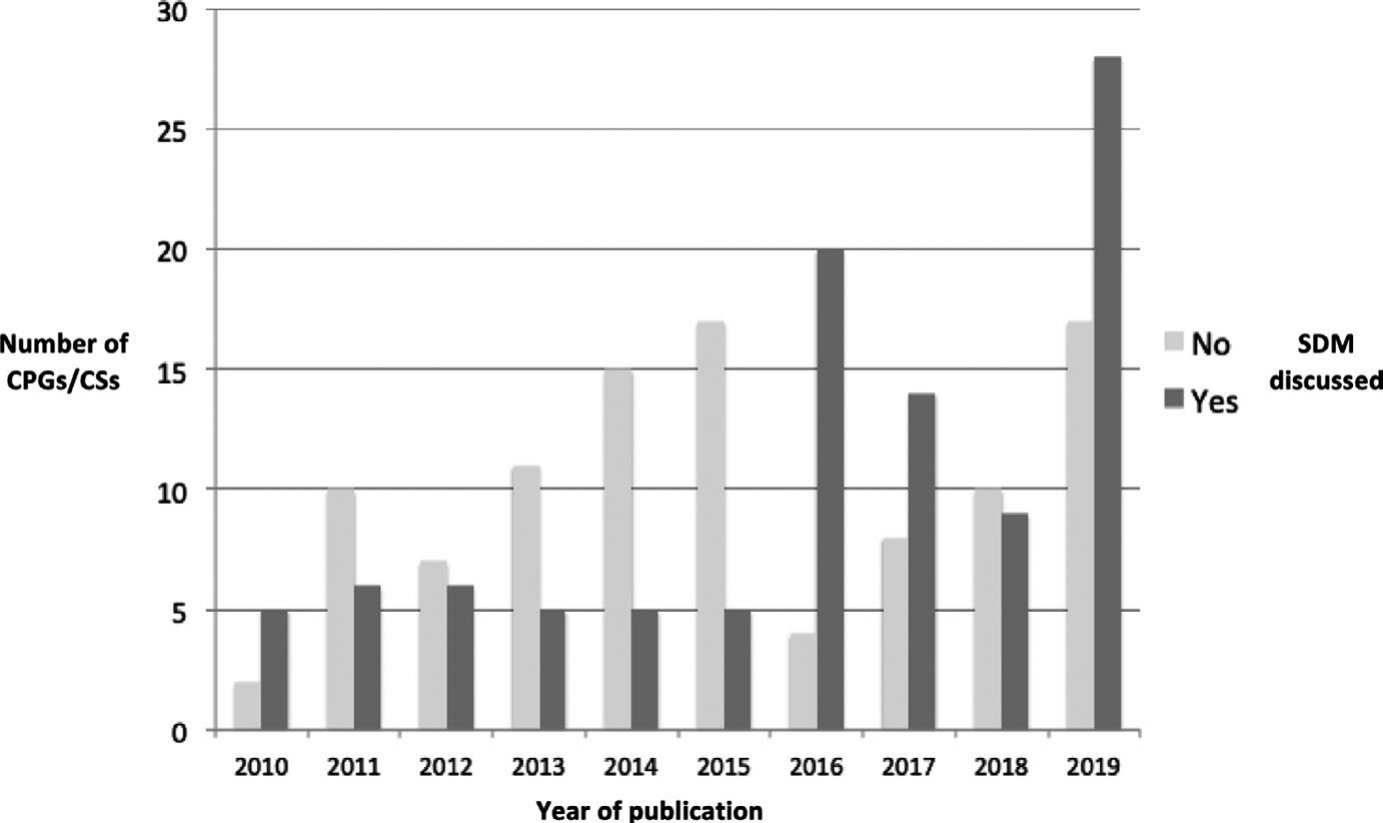
Comparison between the year of publication of the guide according to whether or not SDM appearance

We developed a standardized quality assessment tool for assessing the coverage of SDM in recommendation documents. Our review and analysis showed that SDM description, clarification and recommendations CPGs and CSs concerning BC treatment were poor, leaving a large scope for improvement in this area. SDM more frequently reported in CPGs and CSs in recent years but surprising SDM was less often covered in medical journals (Figure 3).

### 4.2 Strengths and weaknesses

The validity of findings depends on the strength and limitations of methods, which should be understood first before assessing their implications^202^. A key strength of this study was a global perspective with a big number of CPGs and CSs included, without language restrictions or data sources limitations. We developed and deployed a prospective protocol with a specific SDM quality assessment tool incorporating the AGREE II instrument^201^, RIGHT statement^200^ and other related papers ^9^, ^21^, ^24^, ^25^, ^27^. Unfortunately, as there were no other similar studies, we could not compare our results with other findings. There have been evaluations of risk of bias in other papers, but our focus was on examining the reporting of guidance about SDM. One perceived limitation of this study could be related to the subjective nature of the data extraction; however, as we used duplicate data extraction with arbitration, we minimized this methodological issue. Quality assessment tool performance may be a further issue, and we addressed this by following a standard methodology for tool development. Not all quality items can have the same relevance and weight, and future research should focus on scoring them creating a threshold for rating quality. Because the items mainly came from two wide‐used indexes^200^, ^201^, demonstrably our tool should be considered to have face validity. Therefore, we are confident that our finding of poverty of SDM information in practice recommendations is trustworthy and merits further consideration.

Inter‐examiner reliability should be calculated in systematic reviews as the data extracted should be the same by different reviewers^203^. Intra‐examiner reliability is a pre‐condition for inter‐observer reliability, and so was not calculated or reported^31^. In our paper, the inter‐examiner reliability score was found to be excellent (ICC = 0.97).

### 4.3 Implications

To our knowledge, information and recommendations about SDM in BC CPGs and CSs have not been systematically analysed previously. Neither did we find a tool to evaluate SDM reporting quality. This is surprising because SDM is a legal obligation^11^, ^12^, ^13^ and a key component for high‐quality patient‐centred cancer care^6^, ^7^, ^8^, ^9^, ^10^.

Breast cancer is the paradigm of the situation where a two‐way exchange not only of information but also of treatment preferences is needed to find the best option for a particular patient, as different strategies may show a priori similar advantages and disadvantages but possible outcomes are deeply related to the patient’s values and personal situation^10^, ^203^.

Formal recommendations should promote SDM application in clinical routine practice, but this has proved difficult and slow^18^, ^19^, ^20^, ^21^, ^23^, ^24^. It would require changing attitudes, acquiring new skills, developing specific tools and ensuring an environment where communication and sharing perspectives are valued^10^, ^21^, ^25^, ^26^, ^27^. Effective implementation strategies could be underpinned by SDM detailed in CPGs and CSs as these documents should be expected to provide this specific content^11^, ^12^, ^13^. Our work has identified a gap that offers an important contribution in directing further research and debate, including assessment of risk of bias in guidelines. It highlights the need for more objective‐specific tools for SDM assessment, evaluation of their psychometric properties and promotion in CPGs and CSs for diverse malignancies. Future studies should be required in that direction.

## 5 CONCLUSIONS

This systematic review found that BC treatment CPGs and CSs insufficiently addressed SDM. Implementation of this practice is important for high‐quality patient‐centred cancer care, but lack of knowledge is a known barrier. SDM descriptions and recommendations in CPGs and CSs concerning BC treatment need improvement. SDM was more frequently reported in CPGs and CSs in recent years, but surprisingly it was less often covered in medical journals, a feature that needs attention. In the future, SDM should be suitably explained and encouraged and specific tools should be applied to assess its dealing and promotion in specific cancer treatment CPGs and CSs. Medical journals should play a strong role in promoting SDM in CPGs and CSs they publish in the future.

## CONFLICTS OF INTEREST

The study was conducted in Granada, Spain. There are no conflicts of interest.

## AUTHOR CONTRIBUTIONS

Each author certifies that he/she has made a direct and substantial contribution to the conception and design of the study, development of the search strategy, the establishment of the inclusion and exclusion criteria, data extraction, analysis and interpretation. MMC was involved in the design of the study, literature search, data collection and analysis, quality appraisal and writing. IMMN was involved in the literature search and data collection. MMD was involved in the design of this study, analysis of data and writing. LM was involved in writing. KSK was involved in the design of this study, conducted the quality appraisal, in the writing, and provided critical revision of the paper. ABC was involved in the design of this study and provided critical revision of the paper. All authors read and provided the final approval of the version to be published.

## Supporting information

Appendix S1

Appendix S2

Appendix S3

Appendix S4

Appendix S5

## Data Availability

The authors confirm that the data supporting the findings of this study are available within the article and its supplementary materials.
