# Supplementary material for: Shared decision making in breast cancer treatment guidelines: Development of a quality assessment tool and a systematic review
Source: Health Expect. 2020 Aug 3;23(5):1045–64. doi: 10.1111/hex.13112 (PMC7696137; doi:10.1111/hex.13112)
Supplement: Supplementary file 2 — Appendix S2 [file HEX-23-1045-s002.docx]

**Appendix 2**

**Data sources and search strategy**

**A2.1 Sample search strategy for Medline**

We conducted a systematic search on 15 December 2019 in MEDLINE (via PubMed; from January 2010 to December 2019) using the following combination of free-text terms:

#1 Practice guideline [pt]

#2 Practice guidelines as topic [mesh]

#3 Guideline [pt]

#4 guidelines as topic [mesh]

#5 consensus [mesh]

#6 OR #1-#5

#7 breast neoplasms [mesh]

#8 breast neoplasms [mesh]

#9 breast neoplasms [all]

#9 breast cancer [all]

#10 OR #7-9

#11 2010 [pdta] : 3000[pdta]

# #6 AND # 10 AND #11

Results: 1907 articles

**A2.2 Online databases**

1. MEDLINE
2. EMBASE
3. Web of Science
4. Scopus
5. The Cochrane Database of Systematic Reviews
6. Cochrane Methodology Register
7. ACP Journal Club
8. Database of Abstracts of Reviews of Effects
9. Cochrane Central Register of Controlled Trials (CENTRAL)
10. The Health Technology Assessment

**A2.3 Guideline-specific databases**

1. NHMRC, Australia
2. CMA Infobase, Canada
3. CPG, Canada
4. GIN, International
5. NZGG, New Zealand
6. NICE, UK
7. Trip Database, UK
8. SIGN, UK
9. Fisterra, Spain
10. HSTAT, USA
11. NCCN, USA
12. NGC, USA

**A2.4 Professional societies**

1. Australian Government, Australia
2. KCE, Belgium
3. Alberta Health Services, Canada
4. BCMA, Canada
5. CancerCare Manitoba, Canada
6. CCO & Ontario Ministry of Health, Canada
7. Ministerio de Salud de Chile, Chile
8. Chinese Ministry of Health, China
9. Chinese expert consensus meeting, China
10. Instituto Nacional de Cancerología, Colombia
11. Dirección de desarrollo de Servicio de Salud, Costa Rica
12. ESMO, Europe
13. ESO, Europe
14. ESTRO, Europe
15. EUSOMA, Europe
16. The European Society of Breast Cancer Specialists, Europe
17. St. Gallen/Vienna, Europe
18. ABC3, Germany
19. AGO, Germany
20. DEGRO, Germany
21. ABSI, India
22. ICMR, India
23. ICON, India
24. ESO, International
25. International expert panel, International
26. NCCP, Ireland
27. Japanese Breast Cancer Society, Japan
28. Lithuanian oncologist, encrinologist and General practicioners, Lithuania
29. Richtlijnendatabase, Netherlands
30. SCAN, Singapore
31. FESEO, Spain
32. SEGO, Spain
33. SEOM, Spain
34. ABS, UK
35. BAPRAS, UK
36. Joint Guidelines from British Surgical Associations, UK
37. The Royal College of Radiologist, UK
38. Scottish Cancer Taskforce, UK
39. American Board of Internal Medicine's, USA
40. American Brachytherapy Society, USA
41. American Society of Plastic Surgeons, USA
42. American Society for Radiation Oncology, USA
43. ABS, USA
44. ACOG, USA
45. ASBrS, USA
46. ASBS, USA
47. ASCO, USA
48. ASTRO, USA
49. SSO, USA
50. Society of Surgical Oncology Breast Disease, USA
51. Ministry of Health from New Zealand, New Zealand
