# Supplementary material for: Shared decision making in breast cancer treatment guidelines: Development of a quality assessment tool and a systematic review
Source: Health Expect. 2020 Aug 3;23(5):1045–64. doi: 10.1111/hex.13112 (PMC7696137; doi:10.1111/hex.13112)
Supplement: Supplementary file 3 — Appendix S3 [file HEX-23-1045-s003.docx]

**Appendix 3:**

**Quality assessment tool for Shared Decision Making (SDM) recommendations in Breast Cancer Management Clinical Practice Guidelines (CPG) and consensus (CS)**

| Domain |  | Item | Source of the item | |
| --- | --- | --- | --- | --- |
|  |  |  | AGREE II^(1)^ | RIGHT^(2)^ |
| Basic information | 1 | SDM appears in some section of the CPG |  |  |
|  | 2 | SDM appears in the Executive Summary |  | √ |
|  | 3 | SDM appears in the table of content |  |  |
|  | 4 | SDM appears in glossary, abbreviations, acronyms or topic indexes |  | √ |
| Background | 5 | SDM basis (concept, benefits, risks and limitations) are explained |  | √ |
|  | 6 | Primary affected population is well defined | √ | √ |
|  | 7 | Patients subgroups that need special consideration are discuss | √ | √ |
| Evidence selection criteria | 8 | The key (PICO) question related to SDM is specified | √ | √ |
|  | 9 | Details of the strategy used to search for evidence about SDM is reported |  | √ |
| Evidence strengths & limitations | 10 | Study design(s) and methodology limitations are pondered | √ |  |
|  | 11 | Appropriateness/relevance of outcomes are considered | √ | √ |
|  | 12 | Consistency of results across studies are detailed | √ |  |
|  | 13 | Magnitude of benefit versus magnitude of harm is considered | √ |  |
|  | 14 | Certainty of the supporting evidence on SDM is indicated | √ | √ |
| Recommendations | 15 | Clear and precise recommendations on SDM is provided | √ | √ |
|  | 16 | Distinctive recommendations about SDM for important subgroups are separated | √ | √ |
|  | 17 | Strength of recommendations on SDM is indicated |  | √ |
| Facilitators and barriers | 18 | Facilitators to SDM application are described | √ |  |
|  | 19 | Barriers to SDM application are described | √ |  |
| Implementation advice/tools | 20 | Advice on how recommendations about SDM can be applied in practice is provided |  |  |
|  | 21 | Additional materials to support the implementation of SDM are provided | √ |  |
| Resource implications | 22 | Types of cost of SDM implementation that were considered are specified | √ |  |
|  | 23 | Information/description of the cost information is provided | √ |  |
|  | 24 | The information gathered affects recommendations about SDM and it is well detailed | √ |  |
| Monitoring/auditing criteria | 25 | Criteria to assess adherence to recommendations about SDM | √ |  |
|  | 26 | Criteria for assessing impact of implementing these recommendations | √ |  |
|  | 27 | Advice on the frequency and interval of measurement of these criteria | √ |  |
| Recommendations & limitations | 28 | Suggestions for further research are provided based on the gaps in the evidence encountered |  | √ |
|  | 29 | Limitations of the guideline about SDM recommendations are described | √ | √ |
| Editorial Independence & declaration of interest | 30 | Declaration of the value of the SDM use is described |  |  |
|  | 31 | Declaration / management of interests (professional, financial or intellectual) about SDM use is described | √ | √ |
