# Supplementary figures and images for: Shared decision making in breast cancer treatment guidelines: Development of a quality assessment tool and a systematic review
Source: Health Expect. 2020 Aug 3;23(5):1045–64. doi: 10.1111/hex.13112 (PMC7696137; doi:10.1111/hex.13112)

**Appendix 4: Data extraction analysis**


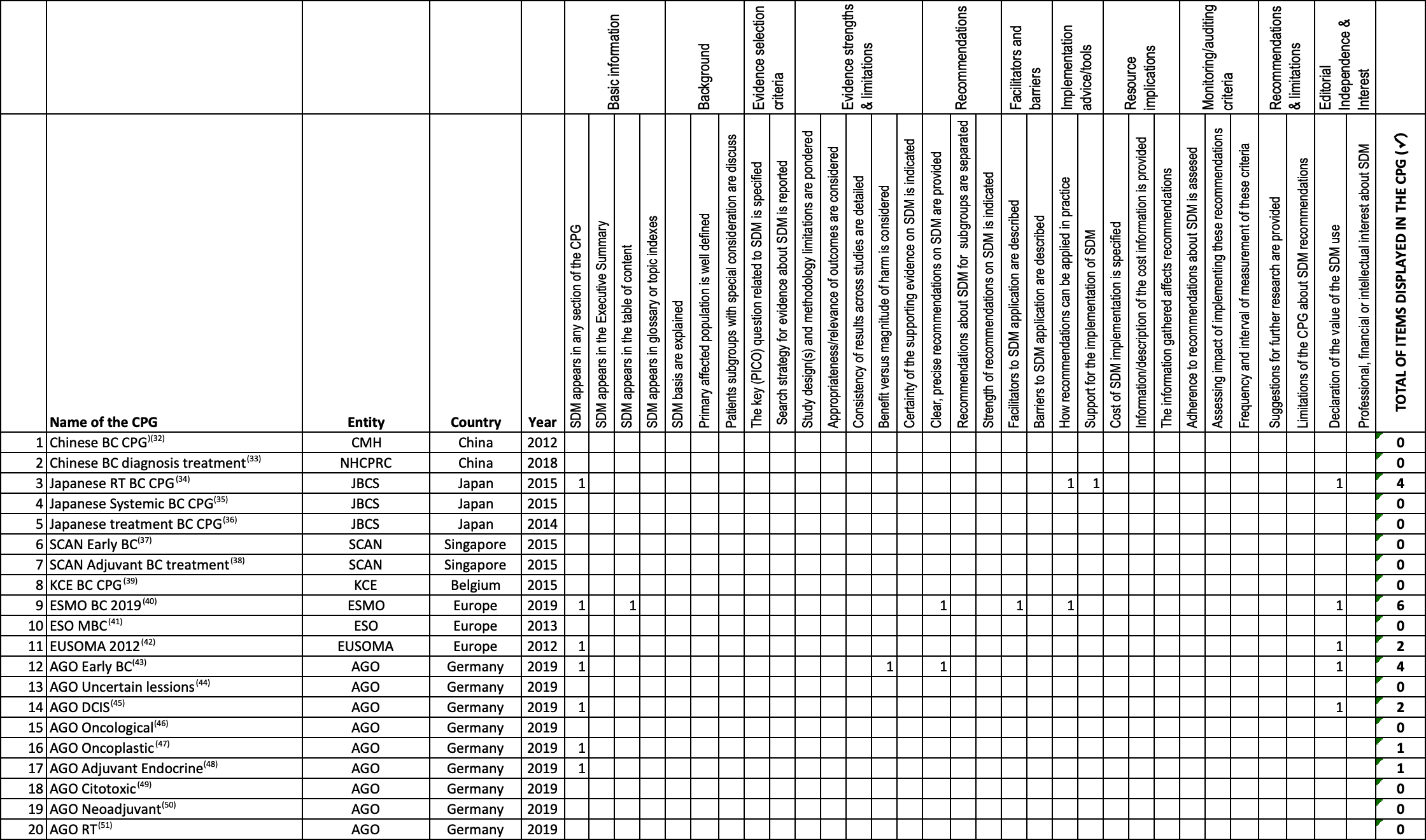


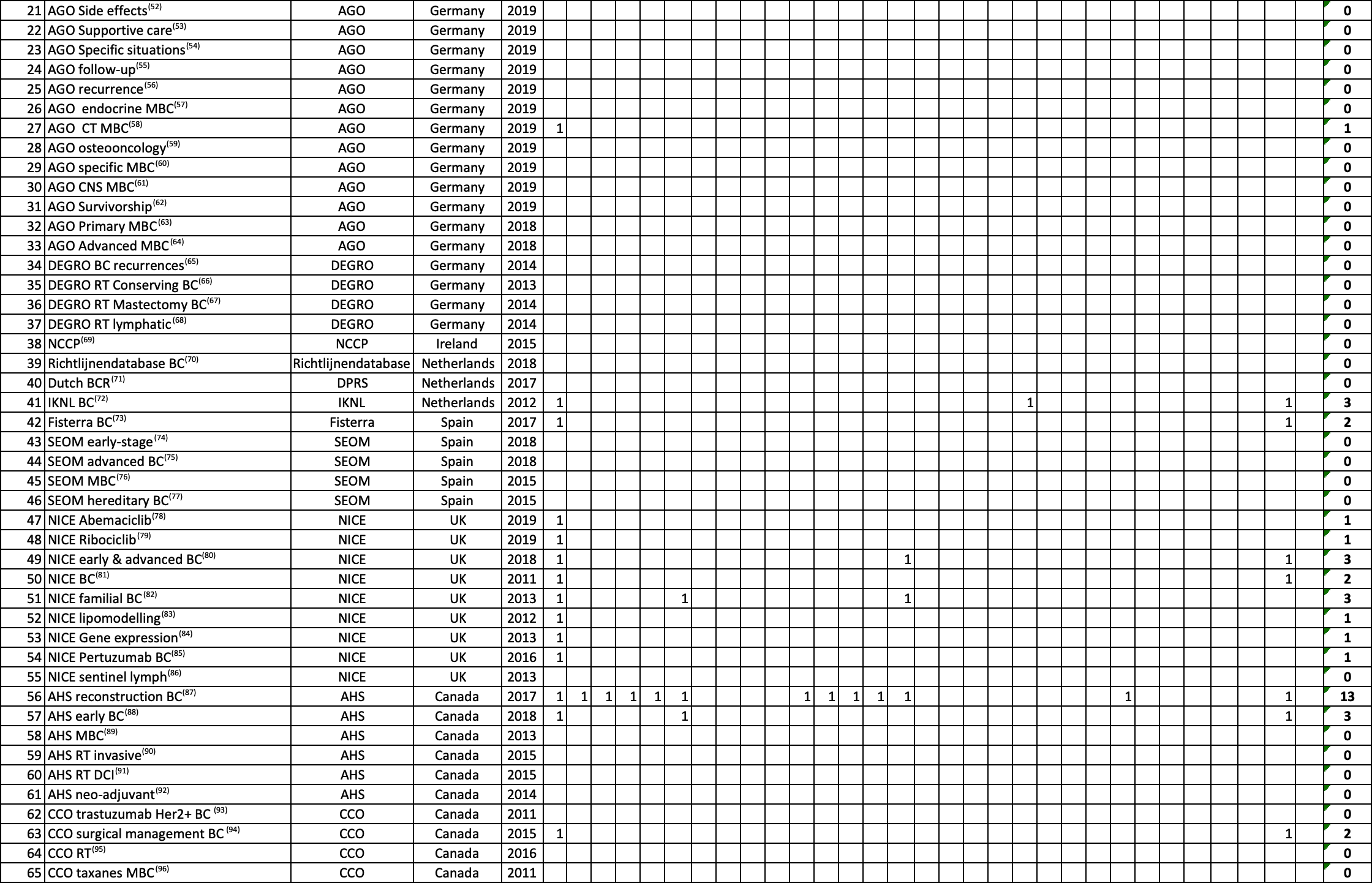


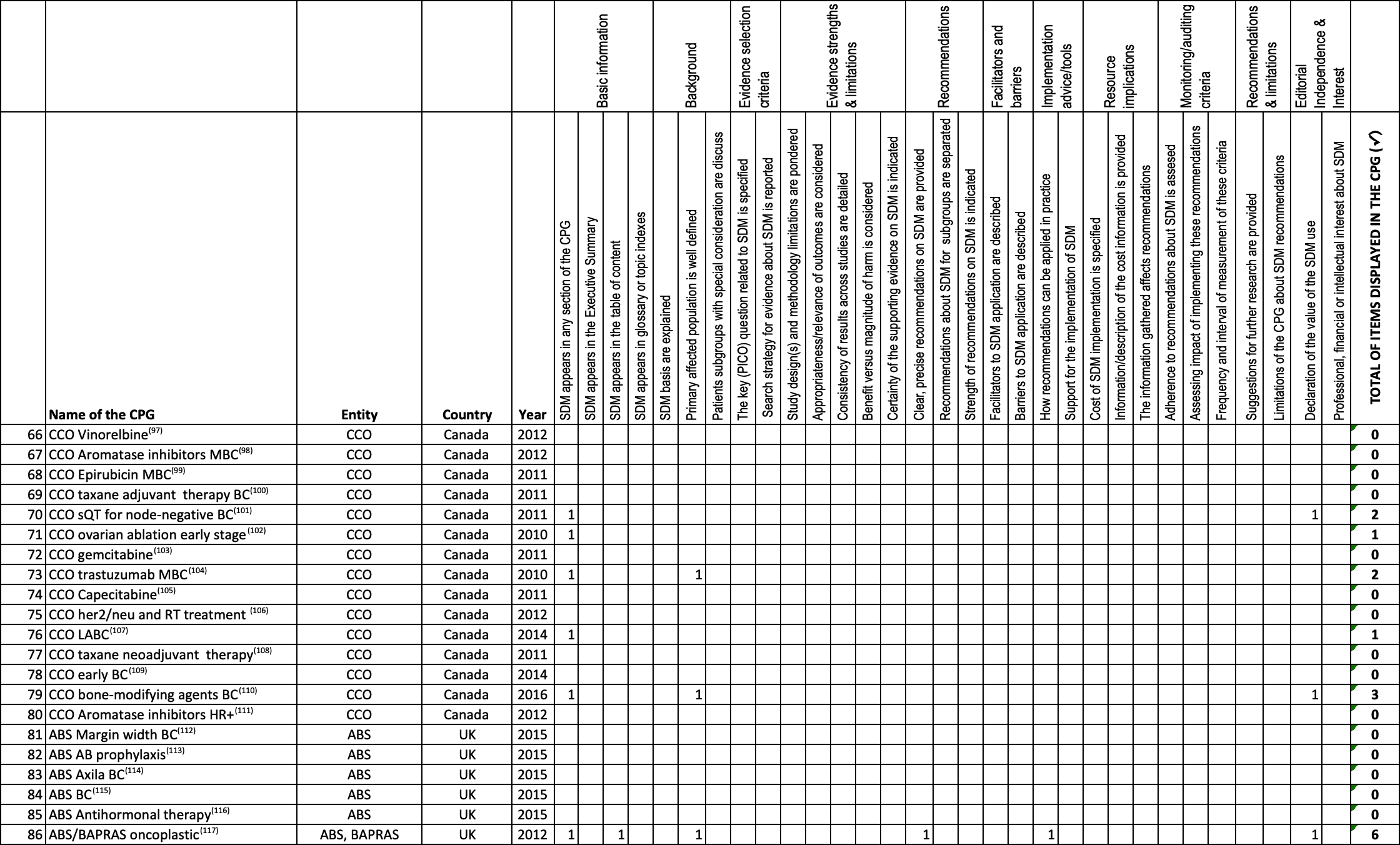


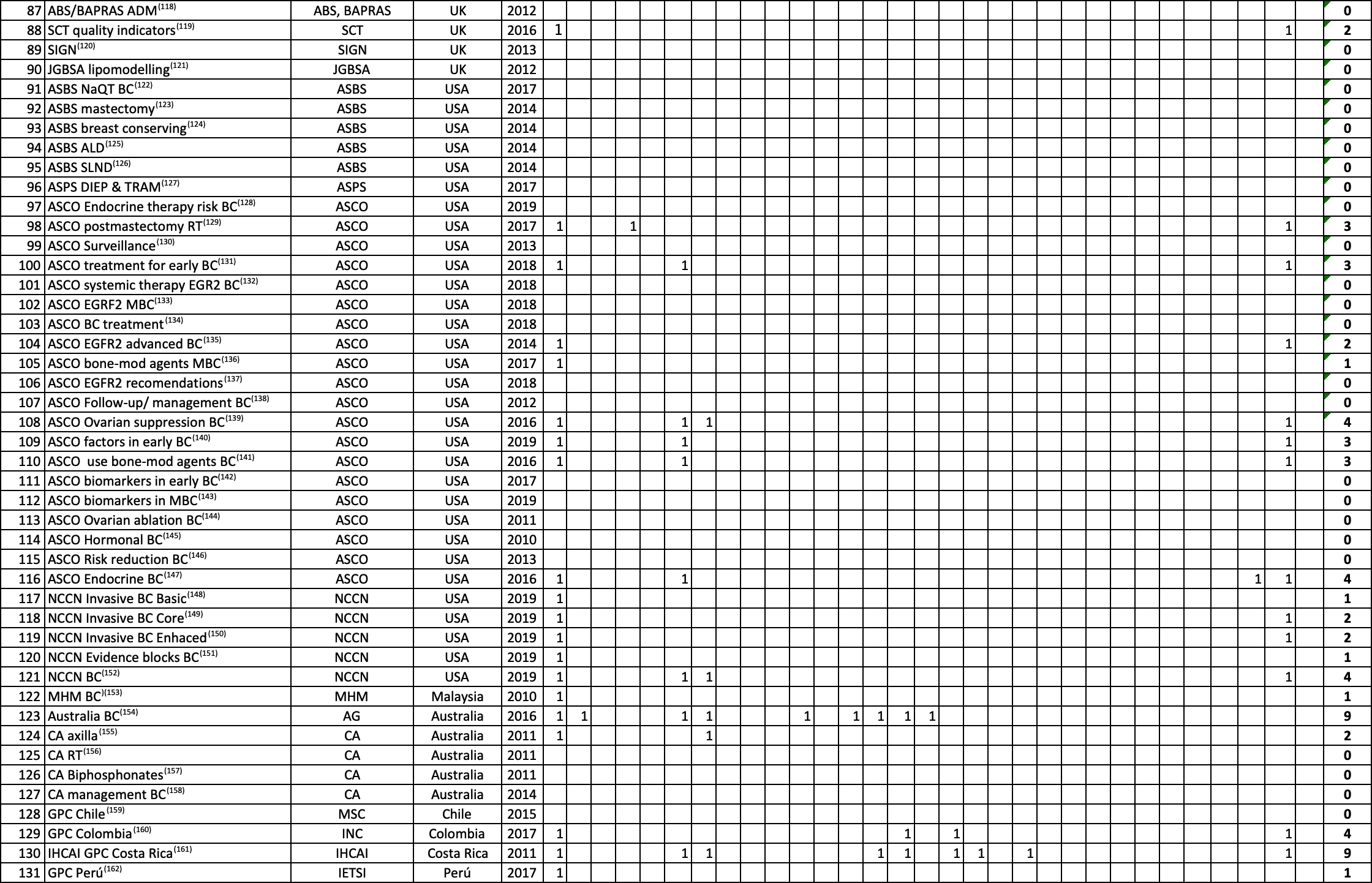


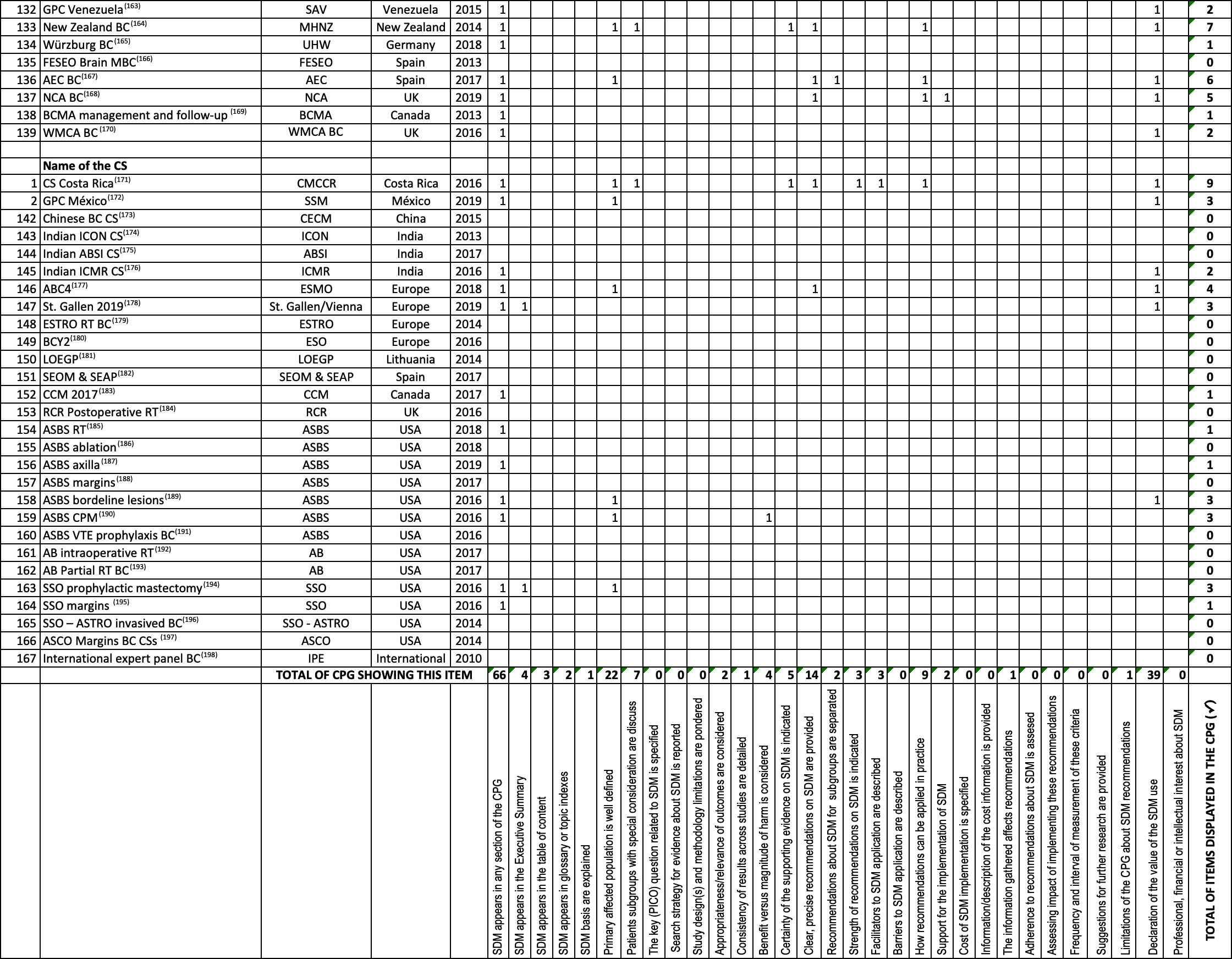

Supplement: Supplementary file 4 — Appendix S4 [file HEX-23-1045-s004.docx]
