# Supplementary material for: Shared decision making in breast cancer treatment guidelines: Development of a quality assessment tool and a systematic review
Source: Health Expect. 2020 Aug 3;23(5):1045–64. doi: 10.1111/hex.13112 (PMC7696137; doi:10.1111/hex.13112)
Supplement: Supplementary file 5 — Appendix S5 [file HEX-23-1045-s005.docx]

Appendix 5: The year and Last updated date (months) of CPGs and CSs analyzing.

Note: CPGs and CS in grey stated SDM.
